# Do regional trade agreements increase technology complexity of digital service export trade? Evidence from digital intellectual property rules

**DOI:** 10.1371/journal.pone.0328769

**Published:** 2025-08-21

**Authors:** Zehui Yu, Lihua Dai, Jingwen Lu, Jia Li

**Affiliations:** 1 School of Economy, Shandong Women’s University, Shandong, China; 2 School of Economics, Shandong Normal University, Jinan, China; KPC Medical College and Hospital, INDIA

## Abstract

Based on the technology complexity data of bilateral digital service exports of 64 representative economies in the world from 2010 to 2021, this paper uses the gravity model to explore the heterogeneous trade effects of Regional Trade Agreements (RTAs) from the perspective of digital intellectual property rules. The results show that the higher the commitment level of digital intellectual property rules in RTAs signed between the contracting parties, the more conducive to the enhancement of the technology complexity of bilateral digital service trade exports, and the trade effect of RTAs is significant. The influence of the depth of digital intellectual property rules on the export technology complexity of digital service trade has heterogeneity in rule categories, economic development level of exporting countries, imitation ability of importing countries, and law enforcement gap between importing and exporting countries. The results of the mechanism test show that the digital intellectual property rules in RTAs can increase the technology complexity of digital service enterprises through the scale expansion effect of two-way FDI and the technological innovation effect of exporting countries. This paper provides policy implications for global digital intellectual property governance and the quality development of digital service trade.

## Introduction

The changes in the world economy and trade pattern have triggered the requirements for global governance reform of international trade. In the absence of effective constraints in multilateral frameworks such as WTO, RTAs have become the main way for countries to export trade governance demands. Since the 1990s, the number of RTAs signed globally has begun to increase rapidly. According to WTO statistics, as of June 2024, the number of RTAs declared by countries to the WTO has reached 640, of which 369 are still in force. Accelerating the implementation of the free trade area strategy and establishing close trade links with trading partners are also important elements of China’s new round of opening-up. The 14th Five-Year Plan explicitly proposes to “implement the promotion strategy of free trade areas and build a globally network of high-standard free trade areas”. At present, China has signed 22 Free Trade Agreements (FTAs) with 29 countries and regions such as Australia, South Korea and ASEAN, and is actively applying to join CPTPP and DEPA. A considerable amount of literature has confirmed the trade creation effect of RTAs [1–4]. Therefore, in the context of the weak recovery of the world economy and the contraction of external demand, how to seize the opportunity of regional trade cooperation to promote foreign trade to continue to “improve quality in a stable manner” has become an important issue to be solved urgently in the current economic society.

According to the *Global Trade Update* released by UNCTAD, global trade shrank by 3% in 2023, but service trade showed resilience, with an increase of 8%. Service export competitiveness plays an increasingly important role in a country’s economic growth. With the continuous penetration of global digital technology, the dominant position of digital service trade in service trade has gradually emerged. Countries have seized the historical opportunity of digital economic development to strive to improve the competitiveness of digital service products. According to the findings of Hausmann et al. [5], export technology complexity can describe the technological content of a country’s export products and reflect the country’s export strength. As an effective indicator to measure the quality of a country’s trade, export technology complexity has received extensive attention from domestic and foreign scholars. As the development of digital service trade enters the fast track, the study of the export technology complexity of digital service trade has become particularly important. It is of great significance to improve the export structure of service trade and promote the high-quality development of service trade.

In recent years, the service sector has become a priority area for countries in opening up their economies. High-standard service trade rules are included in RTAs. The main approach of the early literature is to test the overall welfare effect of trade agreements by using a dummy variable for whether or not a country has signed an RTA. However, with the increasing number of RTAs, the content of the agreements are more complex and diverse, and there are great differences in the content of the articles included. Baier et al. [6] have found that there are non-negligible differences between RTAs. Therefore, the impact of the heterogeneity of the articles should be fully considered. With the rapid development of knowledge-intensive digital service trade, digital intellectual property rules have become one of the core issues in the reconstruction of international economic and trade rules [7]. Countries are increasingly including digital intellectual property articles in RTAs signed, and the standards of digital intellectual property rules have also been continuously upgraded. Articles such as “reproduction rights in electronic form”, “government use of software”, and “source code” continue to emerge. For example, the Comprehensive and Progressive Agreement for Trans-Pacific Partnership (CPTPP) provides that “No contracting party shall require the transfer of, or access to, the source code of software owned by a person of the other contracting party as a condition for the import, distribution, sale or use of such software, or of products containing such software, within its territory.” This source code article reflects the CPTPP agreement’s emphasis on protecting the intellectual property rights of software owners. Therefore, focusing on the digital intellectual property rules in RTAs, this paper verifies whether the different depths of digital intellectual property rules in RTAs can help to improve the export technology complexity of digital service trade. It can provide an interpretation of the intellectual property perspective for the high-quality development of digital service trade, and also help to provide decision-making references for the reconstruction of global economic and trade rules.

The possible marginal contributions of this paper are as follows: First, this paper attempts to explain the heterogeneity of trade effects of RTAs from the perspective of digital intellectual property rules. And it distinguishes the types of rules, the level of economic development of exporting countries, the imitation ability of importing countries and the gap in law enforcement to examine the heterogeneous impact of digital intellectual property rules, which enriches the relevant research on the trade effects of RTAs. Secondly, this paper discusses the impact of RTAs on trade “quality” rather than trade scale, by taking the export technology complexity of bilateral digital services as the research object, and provides more empirical evidence for the promotion of high-quality regional economic and trade cooperation. Thirdly, this paper theoretically expounds and empirically tests the internal mechanism of digital intellectual property rules affecting the export technology complexity of digital service trade, in order to provide profound insights into the basic logic of digital intellectual property rules affecting export technology complexity, which will be helpful to provide decision-making references for the reconstruction of global economic and trade rules and the governance of digital intellectual property rights.

The remainder of this paper is structured as follows: The literature review section reviews relevant literature of the depth of RTAs, the technology complexity of exports and the trade effects of RTAs. Scholars from domestic and foreign have conducted few studies on directly exploring the impact of deep RTAs on the technological complexity of exports. The digital intellectual property rules in RTAs section introduces and sorts out the rules on digital intellectual property rights in the framework of RTAs. They can be categorized into 3 groups, including rules related to digital copyright protection, rules related to digital technology protection and rules related to digital intellectual property enforcement protection. The theoretical analysis and research hypothesis section analyzes the channels through which digital intellectual property rules in RTAs affect the technological complexity of exports of digital service trade, and presents the research hypotheses based on the theoretical analysis. The empirical analysis section details the empirical analysis, including benchmark regression, robustness test, endogeneity test, heterogeneity analysis and mechanism test. The result of benchmark regression shows that a higher level of commitment to digital intellectual property rules in RTAs is conducive to increasing the technological complexity of bilateral digital services trade exports. The results of heterogeneity analysis show that different categories of digital intellectual property rules have different impacts on the technological complexity of exports of digital service trade. The stronger the imitation capacity of the importing country, the greater the facilitating effect of digital intellectual property rules on the technological complexity of exports of digital service trade. The larger the enforcement gap between importing and exporting countries, the more the high-standard digital intellectual property rules promote the technological complexity of exporting digital service trade. The results of the mechanism test suggest that digital intellectual property rules in RTAs can increase the export technological complexity of digital services firms through the two-way FDI scale expansion effect and technological innovation effect in exporting countries. The conclusions and recommendations section is the conclusions and related recommendations part of this paper.

## Literature review

Based on the study of the existing literature, there are three main categories of literature that are highly related to this paper, including research on the depth of RTAs, research on the technological complexity of exports and research on the trade effects of RTAs.

### Research on the depth of RTAs

The first category of relevant literature is studies on the depth of RTAs. Since, Lawrence [8] introduced the concept of deep RTAs, many scholars have begun to focus on the study of the depth of RTAs. Horn et al. [9] creatively divided the 52 high-frequency clauses into “WTO+” and “WTO-X” by analyzing the topics covered by the trade agreements signed by the EU and the United States. This method is not only used by WTO as an alternative indicator to measure the depth of preferential trade arrangements, but also used by many other scholars in their research. Dür et al. [10] measured the depth based on seven key articles in trade agreements, from simply including a chapter in a trade agreement to coding the details. Kohl et al. [11] constructed a database containing 296 trade agreements, and constructed the WTO+ coverage index, the WTO-X coverage index and the institutional quality coverage index, respectively. Based on the method of Horn et al. [9], Hofmann et al. [12] further studied the legal enforceability of RTAs articles, and assigned values according to the coverage and legal binding force of the articles to measure the depth of RTAs. The above research has built a rich research foundation for the identification of RTAs depth, but they cannot identify the dynamic development of trade agreements themselves. Therefore, Tie et al. [13] constructed the deepening indicators of RTAs based on the perspective of article coverage and agreement reformulation, expanding the measurement from static to dynamic. As the standards of RTAs continue to improve, the content of the articles covered extends from border measures to post-border measures, and scholars have begun to pay attention to the depth of behind-the-border measures. Zhang [14] divided “core articles” into “border” and “behind-the-border”, and classified state-owned enterprises, competition policy, intellectual property rights, etc. as behind-the-border measures articles, and constructed a depth index based on the legal effect of the articles.

### Research on the export technology complexity

The second category of literature that is highly related to this paper is research on the technological complexity of exports.The export technology complexity indicates a country’s export technology level, which can largely reflect the competitiveness of a country’s export products in the world. Many scholars have focused on the influencing factors of export technology complexity, and conducted research from the perspectives of foreign direct investment [15], infrastructure construction [16], financial development [17], and trade liberalization [18]. The research on the impact of trade liberalization on the export technology complexity is closely related to this paper. Dai [19] conducted an empirical test on the basis of services trade liberalization measured by FDI penetration and import penetration. The results found that the development of service trade liberalization has a significant positive impact on improving the technology complexity of China’s manufactured exports. Cai and Li [20] used cross-country panel data from 40 countries or regions to conduct empirical analyses. The results showed that the import of producer services has a significant positive impact on the improvement of the technology complexity of a country’s service trade export, and there is a large sectoral heterogeneity. Sheng and Mao [18] verified the impact of import trade liberalization on the export technology complexity of China’s manufacturing industry at both the enterprise and industry levels. The results showed that import trade liberalization has significantly improved the export technology complexity of enterprises, and has also significantly promoted the overall export technology complexity of the manufacturing industry. Zhou and Hong [21] examined the differential impact of different types of imports, such as capital goods and intermediate goods, on the export complexity of enterprises. The study found that importing capital goods can increase the export complexity of Chinese enterprises, while importing intermediate goods can inhibit the export complexity of enterprises. Yu et al. [22] examined the impact of digital product imports on the export technology complexity of enterprises on the basis of a comprehensive identification of digital products. The study found that digital product imports can significantly increase enterprises’ export technology complexity by enhancing enterprise productivity and promoting export product diversification.

### Research on the trade effect of RTAs

The third category of literature related to this paper is research on the trade effects of RTAs. Trade effects have always been dominant in the welfare analysis and policy debate of FTAs [23]. Since Viner [24], domestic and foreign scholars have carried out long-term research on the trade effects of FTAs. Scholars generally believe that the conclusion of RTAs is conducive to regional economic and trade cooperation between countries and increasing trade exchanges. By studying the regional economic integration agreements in the Americas, Baier et al. [25] found that the impact of regional economic and trade agreements on trade may have been underestimated before, and the trade creation effect is very significant. Guillin’s [26] study was mainly based on the data of OECD countries. The results showed that the higher the level of regional service trade liberalization, the more conducive to the development of service trade within the trade area. The research of Lin and Bao [3] showed that trade agreements with higher levels of openness have stronger positive promotion effects on gross service value and value-added exports. Yang and Ai [27] found that RTAs can enhance the value-added trade linkages between economies by reducing trade costs and facilitating technology spillovers. Mattoo et al. [28] used the trade gravity model to verify that the deepening of RTAs is conducive to increasing the trade volume among member countries. The research of Zhou et al. [29] showed that the establishment of China-ASEAN Free Trade Area significantly reduces the uncertainty of regional trade policy and significantly increases the productivity of Chinese export enterprises to ASEAN.

Some scholars have also focused on the relevant rules of intellectual property protection in RTAs. Han et al. [30] argued that FTAs with intellectual property protection rules make China import and export more intellectual property-intensive products, with a significant trade creation effect. Campi and Dueñas [31] believed that the signing of trade agreements containing intellectual property protection rules can promote the exports of developed countries, and there is a significant lag effect. On the basis of measuring the scale of bilateral digital content trade, Zhou et al. [7] analyzed the trade effect of digital intellectual property rules in RTAs. The study found that the in-depth improvement of digital intellectual property rules can significantly promote bilateral digital content trade. Dai and Sun [32] used natural language text processing analysis method to quantify the depth of intellectual property protection of FTAs in the Asia-Pacific region. The results showed that the strengthening of the depth of intellectual property protection of FTAs has significantly improved the quality of export products of each country. Based on the text analysis of RTAs signed by China, Sun et al. [33] examined the impact of intellectual property protection rules on the quality of export products. The results showed that the depth of the rules is conducive to improving the quality of China’s export products, and the R&D effect of importing countries is an important transmission channel.

Through the review of the above literature, it can be found that domestic and foreign scholars’ relevant research on RTAs and export technology complexity provides a good reference for the study of this paper. However, there is still less literature that directly explores the impact of RTAs on export technology complexity. The research on this issue is not only of great value to the study of the influencing factors of bilateral export technology complexity of trading partners, but also of great significance for understanding the trade effects of RTAs. Although some scholars have considered the heterogeneous impact of RTAs, the current empirical research on digital intellectual property rules in RTAs is extremely limited. Most of them focus on the impact of digital intellectual property rules on trade scale, lacking attention to trade “quality”. Moreover, the existing literature on the export technology complexity is more from the perspective of export countries, and less from the perspective of bilateral countries. In view of this, this paper takes the export technology complexity of bilateral trade partner countries’ digital services trade as the research object, and focuses on the digital intellectual property rules to explore the trade effect of RTAs.

### Digital intellectual property rules in RTAs

In recent years, the global governance of digital trade has gradually transitioned to the level of RTAs driven by developed economies such as Europe and the United States. This paper argues that digital intellectual property rules are closely related to digital trade. With the rapid development of digital trade, the protection scope and standards of digital intellectual property rules under the framework of RTAs have been developing constantly. At the same time, the claims of countries in intellectual property protection are also fully reflected in the relevant rules of RTAs. As stipulated in Article 4 “Intellectual Property Rights” of the US-Jordan FTA signed as early as 2000, “Each Contracting Party shall adopt or maintain appropriate laws, regulations, and other measures to require that all government agencies use only authorized computer software”. Article 18.4 of the US-Korea FTA (KORUS FTA), “Copyright and Related Rights”, and Article 20.86 of the US-Mexico-Canada Agreement (USMCA), “Goverment Use of Software”, both involve relevant regulations. As the United States has obvious industrial advantages in the field of software services, these articles are important part of the “American-style” digital intellectual property rules. The “Source Code” clauses have also been an important manifestation of the high standard rules of digital intellectual property rights in recent years. Software has the characteristics of high R&D cost and low replication cost, and the software can be copied by obtaining the source code. Therefore, the protection of source code as a trade secret is a common practice in the software trade. However, in order to achieve specific public policy objectives, some countries may use the source code transfer as a condition for market access. Therefore, source code issue has gradually become an important topic in international economic and trade negotiations, and the standards have been gradually raised. For example, the source code clause in CPTPP stipulates that the clause is limited to mass-market software or products containing the software, excluding software for critical infrastructure. The USMCA, on the other hand, further stipulates that the scope of application of the source code clause includes critical infrastructure software, and for the first time proposes to prohibit the transfer of algorithms in source code as a condition for market access.

On the basis of sorting out the relevant RTAs texts and referring to the TAPED database, this paper divides the digital intellectual property rules in RTAs into three categories: the rules related to digital copyright protection, the rules related to digital technology protection, and the rules related to digital intellectual property law enforcement protection. The specific clauses are shown in Table 1.

**Table 1:** Digital intellectual property rules in RTAs.

Rule Categories	Specific Terms
Digital Copyright Protection	Does the agreement include provisions for government use of (non-infringing) software? Does the agreement include provisions for computer-implemented inventions (software patents)? Does the agreement include provisions for the right to reproduce in electronic form in copyright and related rights? Does the agreement include provisions for storage of copyright and related rights works in electronic form?
Digital Technology Protection	Does the agreement include a prohibition on requiring the transfer of, or access to the source code of software owned by individuals as a condition for importing, distributing, selling or using such software? Does the agreement contain provisions for the protection of information rights management (IRM)? Does the agreement contain provisions for the protection of trade secrets or similar undisclosed information/data protection?
Digital Intellectual Property Law Enforcement Protection	Does the agreement comply with the WIPO Internet Treaty? Including the WIPO Copyright Treaty (1996) and the WIPO Performances and Phonograms Treaty (1996). Does the agreement include a list of multilateral agreements related to intellectual property rights? Such as the Rome Convention for the Protection of Performers, Producers of Phonograms and Broadcasting Organizations, and the Protocol Relating to the Madrid Agreement Concerning the International Registration of Marks. Does the agreement contain provisions on the responsibilities of Internet service providers (ISPs)?

Data source: The author collated the data according to the TAPED database (Trade Agreements Provisions on Electronic-commerce and Data) constructed by the University of Lucerne [7].

## Theoretical analysis and research hypothesis

RTAs can encourage contracting parties to reduce service trade barriers to improve the level of service openness of contracting parties, promote the improvement of market system guarantee of digital service trade to reduce the uncertainty risk of digital service trade, and further promote deep cooperation among contracting parties in the field of digital service trade. A survey shows that trade protection caused by incomplete legal rules related to digital intellectual property rights will have an impact on the development of trade [34]. As knowledge-intensive trade, digital service trade is more sensitive to digital intellectual property protection system. The digital intellectual property rules in RTAs are “behind-the-border measures”, which can be made more consistent with the commitments and obligations in RTAs by reforming the domestic regulations of contracting parties. With the deepening of RTAs, improved review clauses and dispute resolution mechanisms can largely ensure that digital intellectual property rights are not infringed, effectively reducing the infringement probability of imitators. At the meantime, the cost of preventing infringement and safeguarding rights for digital service enterprises will also be further reduced. Therefore, it enhances the willingness of enterprises to export high-tech services in order to gain greater competitive advantage in the export market, which in turn improves the country’s export technology complexity.

Based on the above analysis, this study proposes the following research hypothesis:

Hypothesis 1: The digital intellectual property rules in RTAs can help to improve the export technology complexity of digital service trade.

The deepening of RTAs can reduce the threshold of market access and increase the opportunities for enterprises to enter the market, especially for high-end digital service industries such as telecommunications, finance, and insurance. A large number of service providers from abroad can enter the domestic market, and investment exchanges between contracting parties can continue to expand. Standardized rules in the field of digital intellectual property can help simplify intellectual property transactions, increase transparency, and encourage investors to carry out projects in new areas in contracting partner countries. Under the condition of open economy, the larger the OFDI scale of enterprises in exporting countries, the more opportunities to communicate with foreign digital service enterprises in R&D, information services, data input and other aspects, which will bring more international R&D knowledge spillovers [35,36]. The reverse spillover effect of OFDI will undoubtedly contribute to enhance the production efficiency of domestic digital service enterprises, and then improve the technology complexity of digital service export. At the same time, the expansion of IFDI scale in exporting countries can promote the industrial upgrading of digital service trade and enhance the export technology complexity by introducing advanced technology, equipment, management experience and human capital into exporting countries [37,38].

Based on the above analysis, this study proposes the following research hypothesis:

Hypothesis 2: Digital intellectual property rules in RTAs may increase the export technology complexity of digital service trade through the scale expansion effect of two-way FDI in exporting countries.

The rapid development of digital service trade is closely related to the wide application of digital technology. Technological innovation represented by big data and cloud computing enhances the tradability of services, promotes the development of service trade to intellectualization, digitization and networking, which helps to improve the production efficiency and technological level of service-providing enterprises. Intellectual property is an important institutional force to ensure the smooth progress of technological innovation. As a useful supplement to the domestic and foreign intellectual property protection system, the rules of digital intellectual property rights in RTAs largely determine the size of enterprises’ monopoly gains from domestic and foreign innovations, and are the key factors affecting enterprises’ R&D investment and innovation output. Therefore, signing RTAs containing digital intellectual property rules will enable the exporting enterprises of the contracting parties to increase R&D investment and accelerate technological innovation. Innovative products transformed by advanced technology have the advantage of technological innovation and are more conducive to export. That is to say, the further enhancement of technological content in export products is an important manifestation of the improvement of export technology complexity. For digital service export, with the improvement of technological innovation level, the time of technology update in digital service industry is getting shorter and shorter, which has also become an important force to promote the technology complexity of digital service export [39,40].

Based on the above analysis, this paper proposes the following research hypothesis:

Hypothesis 3: Digital intellectual property rules in RTAs may increase the export technology complexity of digital service trade through the technological innovation effect of exporting countries.

## Empirical analysis

### Model setting and data source

Based on the extended gravity model, this study examined the impact of the depth of digital intellectual property rules on the technology complexity of bilateral digital service trade under the RTAs framework. The benchmark regression model is as follows:


$$\begin{array}{cc} n{EXPY}_{ijt}= & \;\alpha_{0}+\alpha_{1}{lnDepth}_{ijt}+\alpha_{2}{Contig}_{ij}+\alpha_{3}{Comlang}_{ij}\;+\alpha_{4}{Col45}_{ij}+\alpha_{5}{lnNet}_{ijt}\;\\ & +\alpha_{6}{Edugap}_{ijt\;}+\alpha_{7}{PGDPgap}_{ijt}+\theta_{it}+\theta_{jt}+\epsilon_{ijt} \end{array}$$ (1)

Among them, i and j represent the exporting country and the importing country, respectively, and t represents the year. ${\text{EXPY}}_{\text{ijt}}$ represents the export technology complexity of digital service trade from country i to country ij in year t. ${\text{Depth}}_{\text{ijt}}$ is the explanatory variable, which is expressed by the depth level of digital intellectual property rules in country i and country j in year t. The control variables include whether the exporting country i and the importing country j are bordered (${\text{Contig}}_{\text{ij}}$), whether they have a common official language (${\text{Comlang}}_{\text{ij}}$), whether they have a colonial relationship (${\text{Col45}}_{\text{ij}}$), the level of Internet penetration (${\text{Net}}_{\text{ijt}}$), the difference in education level (${\text{Edugap}}_{\text{ijt}}$) and the difference in economic development level (${\text{PGDPgap}}_{\text{ijt}}$). $\theta_{\text{it}}$ and $\theta_{\text{jt}}$ represent the fixed effects of exporter-year and importer-year, respectively, which are used to control the multilateral resistance terms of exporting and importing countries and other time-varying national characteristics (such as their respective GDP, population size, etc.). And $\epsilon_{\text{ijt}}$ is a random disturbance term. The specific meanings of the variables and the sources of the data are shown in Table 2.

**Table 2:** Explanation of main variables.

Variables		Explanation	Data Source
Explained variable	lnEXPY	Export technology complexity of bilateral digital service trade, taking a logarithmic form.	OECD Database
Explanatory variable	lnDepth	A depth index of digital intellectual property rules, taking a logarithmic form.	TAPED Database
Control variables	Contig	Dummy equal to 1 if countries are contiguous.	CEPII Gravity Database
	Comlang	1 if countries share common official or primary language.	CEPII Gravity Database
	Col45	1 if countries are or were in colonial relationship post 1945.	CEPII Gravity Database
	lnNet	The level of Internet penetration in import and export countries, to measure the level of Internet infrastructure construction of both trading parties, taking the logarithmic form of the sum of the Internet penetration rates of importing and exporting countries.	World Bank Database
	Edugap	Difference in education level between import and export countries, taking the logarithmic form of the difference in education expenditures between exporting and importing countries.	World Bank Database
	PGDPgap	Difference in economic development level between import and export countries, taking the difference of the logarithmic form of per capita GDP between exporting and importing countries.	World Bank Database

This study selected the export technology complexity of bilateral digital service trade of 64 economies from 2010 to 2021 as the research object, which are shown in Table 3. The sample economies are selected on the basis of the top-ranking digital service trading countries and their major trading partners in the world, which is representative. The sample countries cover countries in Asia, Europe, the Americas, Oceania, and Africa, ensuring the comprehensiveness and balance of the research objects and minimizing selective bias.

**Table 3:** Sample economies.

Region	Number of Countries	Name of Countries
Asia	17	Armenia, Bahrain, Brunei, China, India, Indonesia, Jordan, Japan, Kazakhstan, Kyrgyzstan, Republic of Korea, Malaysia, Philippines, Singapore, Thailand, Tajikistan, Vietnam
Europe	33	Austria, Belgium, Bulgaria, Belarus, Switzerland, Czech Republic, Germany, Denmark, Spain, Estonia, Finland, France, United Kingdom, Greece, Croatia, Hungary, Ireland, Italy, Lithuania, Luxembourg, Latvia, Moldova, Netherlands, Norway, Poland, Portugal, Romania, Russia, Serbia, Slovak Republic, Slovenia, Sweden, Ukraine.
North America	4	Canada, Mexico, United States, Guatemala
Central and South America	5	Chile, Colombia, Costa Rica, Peru, Panama
Oceania	2	Australia, New Zealand
Africa	3	Egypt, Morocco, South Africa

#### Explained variable.

The explained variable in this study is the export technology complexity of bilateral digital service trade, which takes a logarithmic form of ${EXPY}_{ijt}$. This study drew on the experience and practices of Hausmann et al. [5], Dai [18], and others, to measure the export technology complexity of bilateral digital service trade. First of all, according to this method, in order to calculate the technical complexity of a country or region at the national level, it is necessary to first obtain the technology complexity index (${PRODY}_{kt}$) at the sectoral level of digital service trade, and the calculation formula is:


$${PRODY}_{kt}=\sum_{i}\frac{x_{ikt}/X_{it}}{\sum_{i}\left(x_{ikt}/X_{it}\right)}Y_{it}$$ (2)

Among them, ${PRODY}_{kt}$ is the export technology complexity index of digital service department k in year t. $x_{ikt}$ is the export volume of country i in department k in year $\begin{array}{c} t \end{array}$, $X_{it}$ is the total export volume of digital services of country i in year t. $Y_{it}$ represents the per capita income level of country i in year t, which is expressed by per capita GDP.

On the basis of equation (2), the export technology complexity of bilateral digital service trade is measured using the bilateral country-level export structure as the calculation weight (${EXPY}_{ijt}$):


$${EXPY}_{ijt}=\sum_{k}\frac{x_{ijkt}}{X_{ijt}}{PRODY}_{kt}$$ (3)

Among them, ${\;x}_{ijkt}$ represents the export volume from country i to country j in department k in year t, and $X_{ijt}$ represents the total export volume of digital services from country i to country j in year t. The final results are taken in logarithmic form ($\text{ln}{\text{EXPY}}_{\text{ijt}}$) to mitigate the problem of heteroskedasticity.

#### Explanatory variable.

The explanatory variable of this study is the depth of digital intellectual property rules ${Depth}_{ijt}$, taking the logarithmic form. As shown in Table 1, this study divided the digital intellectual property rules in RTAs into 10 specific articles and classified them into three different types of sub-rules, namely, digital copyright protection rules, digital technology protection rules, and digital intellectual property law enforcement protection rules. The scores of 10 specific articles were assigned according to the TAPED database constructed by Burri of the University of Lucerne, Switzerland. The TAPED database divides each digital trade article in RTAs into soft, mixed and hard articles according to their legal binding force, with values of 1, 2 and 3 points, respectively. If the article is not included, a score of 0 is assigned. The higher the score, the higher the level of commitment of the contracting party to this article. The depth of digital intellectual property rules in RTAs is calculated by summing the scores of 10 specific articles according to equation (4). The depth values of the three types of sub-rulesare calculated according to the scores of specific articles contained in Table 1.


$$Depth=\sum_{i=1}^{n}Article$$ (4)

Among them, Depth represents the depth of digital intellectual property rules covered in RTAs signed by both trading parties, Article represents the assignment value of a specific article, according to TAPED database, and the score is 0–3, totaling 10 clauses. In this paper, the the value for the year before the import and export parties signed a RTA takes the value of 0, and the value for the year in and after the signing is calculated according to the above formula. If two or more RTAs are concluded between importing and exporting countries at the same time, overlay the depth. The final results are taken in logarithmic form ($\text{ln}{\text{Depth}}_{\text{ijt}}$) to mitigate the problem of heteroskedasticity.

#### Control variables.

[1] The observable trade cost variables between import and export countries, including whether they share a border (${Contig}_{ij}$), a dummy equal to 1 if countries are contiguous. Whether they have a common official language (${Comlang}_{ij}$), equal to 1 if countries share common official or primary language. And whether they have a colonial relationship (${Col45}_{ij}$), equal to 1 if countries are or were in colonial relationship post 1945. They are important variables in traditional gravity models [41,42]. International trade is influenced by distance between countries, cultural background, institutional environment, etc. If two countries are close and have similar cultures and institutions, it means that can reduce the cost of trade between the two countries, facilitating the occurrence of trade, which in turn contributes to the technology complexity of exports. The variables are all derived from the Gravity database of CEPII.
[2] The level of internet penetration in import and export countries (${Net}_{ijt}$), which is used to measure the level of internet infrastructure construction of both trading parties. The level of Internet penetration consists of the sum of the percentages of Internet users in the respective populations of import and export countries, in logarithmic form (${lnNet}_{ijt}=\text{ln}({Net}_{it}+{Net}_{jt})$). The popularization of the internet can promote the flow and sharing of information, making it easier for enterprises and individuals to acquire and share knowledge, technology and market information. This rapid flow of information helps enterprises to understand the dynamics of the international market and technological trends, so that they can adjust their product structures and production strategies and improve the technological content and added value of their export products. The data comes from the World Bank database.
[3] Difference in education level between import and export countries (${Edugap}_{ijt}$). This indicator is measured by the absolute value of the logarithmic difference between the educational expenditure of the import and export countries (${Edugap}_{ijt}={|lnEdu}_{it}-{lnEdu}_{jt}|$). The improvement of education level helps to accumulate human capital, improve technology absorption and transformation capacity, optimize industrial structure and promote the development of high-tech industries. Generally speaking, the wider the gap between the education levels of the two countries, the more unfavorable it is to the improvement of the technology complexity of exports. The data on education expenditure in various countries comes from the World Bank database.
[4] Difference in economic development level between import and export countries (${PGDPgap}_{ijt}$). This indicator is measured by the absolute value of the logarithmic difference between the per capita GDP of the import and export countries (${PGDPgap}_{ijt}={|lnPGAP}_{it}-{lnPGDP}_{jt}|$). The level of per capita income is significantly associated with the promotion of higher consumption capacity, increased R&D investment, optimization of industrial structure, and international trade competitiveness, and is therefore able to influence the technological sophistication of exports [30]. The large gap between the two countries’ income levels indicates that the gap between the two countries in terms of consumption and R&D is also large, which is not conducive to the development of high-level trade. The data on GDP Per capita is obtained from the World Bank database. Descriptive statistics of the main variables are shown in Table 4.

**Table 4:** Descriptive statistics of main variables.

Variables	Observation	Mean	Standard Deviation	Minimum	Maximum
lnEXPY	3,804	10.51	0.622	0	10.88
lnDepth	3,804	3.074	2.151	0	5.587
${lnDepth}^{\text{1}}$	3,804	1.184	1.346	0	3.332
${lnDepth}^{\text{2}}$	3,804	2.081	1.587	0	4.382
$ln{Depth}^{\text{3}}$	3,804	2.760	2.034	0	5.081
Contig	3,804	0.136	0.342	0	1
Comlang	3,804	0.158	0.365	0	1
Col45	3,804	0.0410	0.198	0	1
lnNet	3,804	5.036	0.173	4.103	5.286
Edugap	3,804	1.763	1.311	0.00146	7.050
PGDPgap	3,804	0.956	0.870	2.39e-05	4.185

### Benchmark regression results

Based on the regression equation (1), this study examined the impact of the depth of digital intellectual property rules in RTAs on the export technology complexity of bilateral digital service trade. The benchmark regression results were shown in Table 5. Columns (1) and (2) in Table 5 did not control the fixed effect, and the regression coefficient of the depth of digital intellectual property rules was significantly positive. The fixed effects of exporter-year and importer-year were controlled in Columns (3) and (4). The regression coefficient was 0.0816 when other variables were controlled, which was significantly positive at the level of 1%, that is, the improvement of the commitment level of digital intellectual property rules in RTAs is conducive to the improvement of the technology complexity of bilateral digital service trade exports between contracting partners. Signing digital intellectual property rules can coordinate the institutional environment of bilateral trade and protect digital intellectual property from infringement. It can also promote enterprises in import and export countries to continuously improve their technological level in service trade, thereby increasing the technological complexity of exports. It can be seen that importing and exporting countries can effectively promote the high-quality development of bilateral digital services trade by strengthening intellectual property protection, optimizing the trade environment, and promoting technological upgrading, so as to enhance the technological complexity and international competitiveness of exports [43,44]. In terms of the control variables, the coefficients of common language and Internet penetration level were significantly positive, that is, having a common official language and improving the Internet penetration level of the two countries could promote the export technology complexity of bilateral digital service trade. The regression coefficients of the differences in education level and economic development level were significantly negative, which indicated that the greater the gap between the two countries’ education level and economic development level, the less the export technology complexity of bilateral digital service trade. Colonial relationship and border status had no significant impact on the export technology complexity of digital service trade. Col45and Contig are important variables in traditional gravity models. However, the development of the technological complexity of digital service export trade mainly relies on digital technology, so Col45and Contig have limited influence on it, without significant results. Comlang implies that the trading parties have similar cultural backgrounds, which can lower barriers to digital service trade. Internet penetration is the basis for the development of digital service trade. The higher the level of Internet penetration in the two countries, the more opportunities can be created for the occurrence of digital service trade. It is significant to the export enterprises to improve the technological complexity of trade. The improvement of education and economic level is beneficial to the development of digital technology. Therefore, the big difference between the education and economic level of importing and exporting countries means that the gap between the two sides of the trade in digital technology and complexity of digital service trade structure is bigger, which is not conducive to the improvement of the technological service export trade.

**Table 5:** Benchmark regression results.

Variable	(1)	(2)	(3)	(4)
	lnEXPY	lnEXPY	lnEXPY	lnEXPY
lnDepth	0.0248***	0.0317***	0.0241***	0.0816***
	(0.0064)	(0.0094)	(0.0092)	(0.0313)
Contig		-0.0247***		0.0362
		(0.0061)		(0.0456)
Comlang		0.0853***		0.1250***
		(0.0205)		(0.0462)
Col45		0.1638***		−0.0185
		(0.0349)		(0.0555)
lnNet		0.1308*		3.1087**
		(0.0720)		(1.2238)
Edugap		-0.0424***		−0.0386***
		(0.0142)		(0.0130)
PGDPgap		0.0390***		−0.1702**
		(0.0046)		(0.0726)
Constant	10.4345***	9.7754***	10.4367***	−15.4486**
	(0.0295)	(0.3519)	(0.0339)	(6.1306)
Exporter-year fixed effect	No	No	Yes	Yes
Importer-year fixed effect	No	No	Yes	Yes
Observation	3,804	3,804	3,804	3,804
R^2^	0.0074	0.0204	0.3633	0.3690

Note: *, **, and *** indicate significant at the 10%, 5%, and 1%, respectively. Values in parentheses are robust standard errors.

### Robustness test

#### Replace the explanatory variable.

In the benchmark regression of this study, the calculation of the depth of digital intellectual property rules in RTAs, the explanatory variable, is obtained by summing up the assignments of the 10 specific articles it contains. In order to test the robustness of benchmark regression, this study used the arithmetic average to replace the original explanatory variables for regression, and the results were shown in column (1) of Table 6. The regression coefficient of the explanatory variable was 0.0891, which was significantly positive at the level of 1%. The result indicated that deeper contracting of digital intellectual property rules in RTAs is effective in improving the technological complexity of digital service export trade. And it is generally consistent with the result of the benchmark regression, suggesting that the regression results were robust.

**Table 6:** Results of robustness test.

Variable	(1)	(2)	(3)	(4)
	Replace the Core Explanatory Variable	PPML	2010-2019	Hysteresis Effect
	lnEXPY	lnEXPY	lnEXPY	lnEXPY
lnDepth	0.0891***	0.0051***	0.0969***	0.0609**
	(0.0335)	(0.0017)	(0.0371)	(0.0295)
Constant	−15.4806**	1.3991***	−16.5910**	−12.8351**
	(6.1354)	(0.3315)	(6.4504)	(5.9931)
Exporter-year fixed effect	Yes	Yes	Yes	Yes
Importer-year fixed effect	Yes	Yes	Yes	Yes
Observation	3,804	3,804	3,170	3,487
R^2^	0.3690	0.0035	0.3650	0.3699

Note: *, **, and *** indicate significant at the 10%, 5%, and 1%, respectively. Values in parentheses are robust standard errors.

#### Alternate regression method.

The Poisson pseudo-maximum likelihood estimation method (PPML) is a better choice for dealing with problems of heteroscedasticity and trade zeros in the gravity model [45]. Therefore, this study further used the PPML method for regression, and the results were shown in column (2) of Table 6. The coefficient value of the depth of digital intellectual property rules was significantly positive at the level of 1%, which showed that higher level of digital intellectual property rules commitment can enhance the technological complexity of digital service export trade, and the results obtained in benchmark regression were robust.

#### Change the sample time.

The benchmark regression in this study was based on the data from 2010 to 2021 for empirical analysis. However, the global COVID-19 outbreak at the end of 2019 created new demand for digital services and accelerated the digitalization process of service trade [46]. In order to exclude the digital service trade flow triggered by the epidemic, the data for 2020 and 2021 in the sample were removed and then the regression was performed again. The results were shown in column (3) of Table 6. After eliminating the impact of the epidemic, the digital intellectual property rules had a more significant role in promoting the export technology complexity of digital service trade.

#### Lagging effect.

Generally speaking, it often takes a certain time from the signing of RTAs to their effects, that is, there is often a lagging effect in the impact of RTAs on contracting parties [3,47]. Therefore, this study used the lag period of digital intellectual property rules as the explanatory variable for regression, and the results were shown in column (4) of Table 6. The regression coefficient of the one-phase lag variable was significantly positive at the level of 5%, i.e., the influence of RTAs’ digital intellectual property rules on the export technology complexity of digital service trade will persist, and the regression results were stable.

#### Endogenous test.

Various political and economic factors that affect the contraction of RTAs between countries often have an impact on bilateral trade flows [48], so there may be endogenous problems caused by missing variables. This paper used panel data and added the two-way fixed effects of exporter-year and importer-year to the benchmark regression, which better alleviated the endogenous problem caused by omitted variables. Since other digital trade rules may also affect the export technology complexity, this study further added the RTA dummy variable in column (1) of Table 7. During the sample period, if countries sign a free trade agreement containing digital trade rules in RTAs, the dummy variable takes 1; otherwise, it takes 0. This dummy variable was added to ensure that the trade effect comes from digital intellectual property rules rather than other digital trade rules. The regression results showed that the regression coefficient of the depth of digital intellectual property rules was still significantly positive, and the results were robust.

**Table 7:** Results of endogeneity test.

Variable	(1)	(2)
	Increase signing of dummy variables containing digital trade Dummy variables for rule RTAs	Exclude samples with only bilateral RTAs
	lnEXPY	lnEXPY
lnDepth	0.1018**	0.0713**
	(0.0448)	(0.0309)
RTA	-0.0489	
	(0.0763)	
Constant	−15.3103**	−12.1699**
	(6.1081)	(5.7310)
Exporter-year fixed effect	Yes	Yes
Importer-year fixed effect	Yes	Yes
Observation	3,804	3,612
R^2^	0.3690	0.3718

Note: *, **, and *** indicate significant at the 10%, 5%, and 1%, respectively. Values in parentheses are robust standard errors.

Secondly, to solve the endogenous problems caused by reverse causality, this study referred to the practice [3,49] to exclude the samples that only signed bilateral RTAs between importing and exporting countries for regression. Since bilateral trade may have an impact on the signing of bilateral RTAs, it is unlikely to have a substantial impact on the signing of multilateral RTAs. The signing of multilateral RTAs will be affected by multiple factors and has little to do with bilateral trade. Therefore, the countries that only signed bilateral RTAs were excluded from the sample, and only the influence of digital intellectual property rules in multilateral RTAs on the export technology complexity of digital service trade was examined. As can be seen from column (2) of Table 7, the coefficients of the explanatory variables maintained positive significance.

### Heterogeneity test

#### Heterogeneity analysis based on the category of digital intellectual property rules.

As there are certain differences in the content of digital intellectual property rules, the trade effects of different types of digital intellectual property rules may also be different. As mentioned above, this study divided digital intellectual property rules into three different types of sub-rules, i.e., digital copyright protection rules, digital technology protection rules, and digital intellectual property enforcement protection rules. Columns (1) to (3) in Table 8 showed the regression results that the explanatory variables were the depth of digital copyright protection rules (${Depth}^{\text{1}}$), digital technology protection rules (${Depth}^{\text{2}}$), and digital intellectual property enforcement protection rules (${Depth}^{\text{3}}$), respectively. It can be found that the coefficient of the depth of digital copyright protection rules is 0.0014 and the result is not significant. The coefficients of the depth of digital technology protection rules and the depth of digital intellectual property enforcement protection rules are 0.0843 and 0.0946, respectively, both of which are significantly positive at the 1% level. The results showed that the rules of digital technology protection and digital intellectual property enforcement protection in RTAs significantly promoted the export technology complexity of bilateral digital service trade. However, the impact of digital copyright protection rules on the export technology complexity was not significant. The reasons may be that the content of digital copyright protection rules mainly involve the protection of basic forms of digital service objects, such as electronic storage, replication, software patents, non-infringing software, etc., which have a limited effect on the export technology complexity. On the other hand, the rules of digital technology protection and digital intellectual property enforcement protection, such as source code, the responsibility of internet service providers, etc., have been improved protection degree and implementation difficulty. They have strengthened the binding effect on imitation enterprises and improved the incentive efficiency for independent innovation enterprises, thus significantly improving the export technology complexity of digital service trade. Therefore, when contracting RTAs, the member countries should introduce more relevant provisions on the protection of digital technology and enforcement, such as protection of source code.

**Table 8:** Heterogeneity regression results of digital intellectual property rule categories.

Variable	(1)	(2)	(3)
	lnEXPY	lnEXPY	lnEXPY
$ln{Depth}^{\text{1}}$	0.0014		
	(0.0180)		
$ln{Depth}^{\text{2}}$		0.0843***	
		(0.0321)	
$ln{Depth}^{\text{3}}$			0.0946***
			(0.0351)
Constant	−15.8149**	−15.3841**	−15.4927**
	(6.1550)	(6.1309)	(6.1360)
Exporter-year fixed effect	Yes	Yes	Yes
Importer-year fixed effect	Yes	Yes	Yes
Observation	3,804	3,804	3,804
R^2^	0.3682	0.3689	0.3691

Note: *, **, and *** indicate significant at the 10%, 5%, and 1%, respectively. Values in parentheses are robust standard errors.

#### Heterogeneity analysis based on the level of economic development of exporting country.

Since there are great differences in the level of economic development between developed and developing countries, it is possible that improving the level of intellectual property protection may have a heterogeneous impact on the exports of the two economies. In this study, the samples were divided into two groups: the exporting countries are developed countries and the exporting countries are developing countries according to the economic development level of exporting countries. The results were shown in Table 9. The coefficient of explanatory variables in developed countries was significantly positive at the level of 1%, that is, when the exporting country was developed, the higher the commitment level of digital intellectual property rules in RTAs, the more technology complexity of digital service trade can be improved. The coefficient of the explanatory variable was not significant when the exporting country was a developing country. The possible reason is that the innovation of the knowledge-intensive service industry mainly depends on the “network effect” formed by the good interaction between enterprises, customers, and suppliers [50,51]. As the digital service industry in developing countries has just begun to develop, strengthening the protection of intellectual property rights will increase the cost of use for customers and suppliers, the “network effect” cannot be realized, and the knowledge-intensive service industry cannot be further innovated, thus failing to significantly improve the export technology complexity. When the knowledge-intensive service industry develops to a certain stage, the “network effect” has been formed, and then it is necessary to strengthen intellectual property protection to improve the innovation drive of enterprises. Therefore, for developed countries, signing high-standard digital intellectual property rules is helpful to improve the export technology complexity of digital service trade.

**Table 9:** Regression results for heterogeneity in the level of economic development of exporting country.

Variable	(1)	(2)
	Exporter: developed countries	Exporter: developing countries
	lnEXPY	lnEXPY
lnDepth	0.1139***	−0.0142
	(0.0401)	(0.0155)
Constant	−7.3394	4.6830**
	(6.7312)	(2.0014)
Exporter-year fixed effect	Yes	Yes
Importer-year fixed effect	Yes	Yes
Observation	3,144	660
R^2^	0.4644	0.9808

Note: *, **, and *** indicate significant at the 10%, 5%, and 1%, respectively. Values in parentheses are robust standard errors.

#### Heterogeneity analysis based on the imitation capacity of importing country.

The impact of intellectual property protection on trade may depend on the imitation ability of importing countries [52,53]. In this study, the depth of digital intellectual property rules was multiplied by the dummy variable of the importing country’s imitation ability to form an interactive term, which was included in the model (1) to examine whether there was any heterogeneity caused by the difference of the imitation capacity of the importing countries. The value of the dummy variable referred to Smith’s [52] method. When the R&D expenditure of the importing country accounts for no less than 0.5% of GDP, it is considered as a country with strong imitation ability, and the value is 1, otherwise it is 0. The data on R&D expenditure as a percentage of GDP in various countries comes from the World Bank database. According to the regression results in Table 10, except for the interaction term between the depth of digital copyright protection rules and the imitation ability of the importing country, the coefficients of the interaction terms were significantly positive, indicating that the stronger the imitation ability of the importing country, the greater the promotion effect of digital intellectual property rules on the export technology complexity of digital service trade. The possible reason is that when the importing country has a strong imitation ability, strengthening intellectual property protection will greatly limit the imitation behavior of domestic enterprises in the importing country. The threat of enterprises in the exporting country entering the country’s market will be significantly reduced, and the innovation incentive will be increased, which is more conducive to improving the complexity of export technology.

**Table 10:** Results of regression on heterogeneity of imitation capacity in importing country.

Variable	(1)	(2)	(3)	(4)
	lnEXPY	lnEXPY	lnEXPY	lnEXPY
lnDepth	-0.1490**			
	(0.0716)			
copy×lnDepth	0.2466***			
	(0.0914)			
$ln{Depth}^{\text{1}}$		-0.0519		
		(0.0379)		
$copy\times ln{Depth}^{\text{1}}$		0.0600		
		(0.0425)		
$ln{Depth}^{\text{2}}$			-0.1553**	
			(0.0767)	
$copy\times ln{Depth}^{\text{2}}$			0.2588***	
			(0.0983)	
$ln{Depth}^{\text{3}}$				-0.1672**
				(0.0789)
${copy\times lnDepth}^{\text{3}}$				0.2775***
				(0.1000)
copy	-0.1400	0.0257	−0.2087	−0.1596
	(0.1051)	(0.0775)	(0.1358)	(0.1138)
Constant	−13.8567**	−15.5012**	−13.6513**	−14.0775**
	(5.8640)	(6.1366)	(5.8078)	(5.9255)
Exporter-year fixed effect	Yes	Yes	Yes	Yes
Importer-year fixed effect	Yes	Yes	Yes	Yes
Observation	3,804	3,804	3,804	3,804
R^2^	0.3702	0.3683	0.3700	0.3705

Note: *, **, and *** indicate significant at the 10%, 5%, and 1%, respectively. Values in parentheses are robust standard errors.

#### Heterogeneity analysis based on gaps in law enforcement efforts.

As an important innovation protection system, intellectual property rights require strong law enforcement to ensure that the system works. This study uses the scores of Legal System and Property Rights in the Economic Freedom Index published by the Fraser Institute, a Canadian think tank, to characterize a country ‘s law enforcement efforts, and to explore whether the trade creation effect of digital intellectual property rules in RTAs will change due to the gap in law enforcement between the parties. This indicator, which mainly reflects the ability of the government to protect individuals and their legally acquired property, is considered to reflect the legal implementation of property rights protection in various countries [54]. The law enforcement gap was measured by the absolute value of the logarithm difference between the legal system and property rights scores of the importing and exporting countries (${Legalgap}_{ijt}={|lnLegal}_{it}-{lnLegal}_{jt}|$), and then it was added to the model (1) for regression after interacting with the rules of digital intellectual property rights. According to the results in Table 11, the coefficients of most interactive terms were significantly positive, indicating that the greater the enforcement gap between importing and exporting countries, the more high-standard digital intellectual property rules can promote the export technology complexity of digital service trade. The possible reason is that when there is a big gap in law enforcement between the two sides of the trade, the conclusion of RTAs containing digital intellectual property rules will help narrow the gap between the two countries. Then it can reduce the risks and uncertainties in digital intellectual property protection and create a more stable institutional environment for the development of digital service trade, thus promoting the export of knowledge-intensive products by enterprises and enhancing the export technology complexity.

**Table 11:** Regression Results for heterogeneity in the enforcement gap.

Variable	(1)	(2)	(3)	(4)
	lnEXPY	lnEXPY	lnEXPY	lnEXPY
lnDepth	0.0961***			
	(0.0340)			
Legalgap×lnDepth	0.0487**			
	(0.0237)			
$ln{Depth}^{\text{1}}$		0.0079		
		(0.0181)		
$Legalgap\times {lnDepth}^{\text{1}}$		0.0276		
		(0.0171)		
${lnDepth}^{\text{2}}$			0.0963***	
			(0.0339)	
$Legalgap\times {lnDepth}^{\text{2}}$			0.0466**	
			(0.0234)	
${lnDepth}^{\text{3}}$				0.1125***
				(0.0389)
$Legalgap\times ln{Depth}^{\text{3}}$				0.0506**
				(0.0247)
Legalgap	0.0418	0.0735	0.0451	0.0368
	(0.0806)	(0.0724)	(0.0816)	(0.0818)
Constant	−15.9455**	−14.8124*	−15.6923**	−15.7824**
	(7.7666)	(7.6523)	(7.8438)	(7.6827)
Exporter-year fixed effect	Yes	Yes	Yes	Yes
Importer-year fixed effect	Yes	Yes	Yes	Yes
Observation	3,804	3,804	3,804	3,804
R^2^	0.3700	0.3688	0.3699	0.3702

Note: *, **, and *** indicate significant at the 10%, 5%, and 1%, respectively. Values in parentheses are robust standard errors.

### Mechanism test

The benchmark regression results showed that the digital intellectual property rules under the RTAs framework contributed to the improvement of the export technology complexity of bilateral digital service trade in contracting countries. Hypotheses 2 and 3 respectively pointed out that digital intellectual property rules may enhance the export technology complexity of digital service trade by promoting the scale of two-way FDI and the level of technological innovation in exporting countries. In this study, the mechanism test was carried out following Jiang's method [55], and the model is as follows:


$${lnFDI}_{ijt}=\alpha_{0}+\alpha_{1}ln{Depth}_{ijt}+\alpha_{2}{Contig}_{ij}+\alpha_{3}{Comlang}_{ij}+\alpha_{4}{Col45}_{ij}+\alpha_{5}{lnNet}_{ijt\;}\;+\alpha_{6}{Edugap}_{ijt}+\alpha_{7}{PGDPgap}_{ijt}+\theta_{it}+\theta_{jt}+\varepsilon_{ijt}$$ (5)

$${lnINNO}_{ijt}=\alpha_{0}+\alpha_{1}ln{Depth}_{ijt}+\alpha_{2}{Contig}_{ij}+\alpha_{3}{Comlang}_{ij}+\alpha_{4}{Col45}_{ij}+\alpha_{5}{lnNet}_{ijt}\;+\alpha_{6}{Edugap}_{ijt}+\alpha_{7}{PGDPgap}_{ijt}+\theta_{it}+\theta_{jt}+\varepsilon_{ijt}$$ (6)

Among them, the FDI in model (5) represents the weighted two-way FDI scale of the exporting countries, taking the logarithmic form. Since there are many missing values in bilateral FDI data between countries and the data dimension is mainly exporter-importer-time level data. Therefore, this study uses the proportion of bilateral exports of digital service trade to the total exports of digital service trade in exporting countries as the weight of the sum of OFDI and IFDI stocks of exporting countries. The OFDI and IFDI data of exporting countries are from the UNCTAD database. In model (6), INNO represents the weighted technological innovation level of exporting country, taking the logarithmic form. Referring to the method of Liu and Zhen [56], this study adopted the PCT patent application amount of the exporting country to represent a country’s technological innovation level and also used the proportion of bilateral exports of digital service trade in the total exports of the exporting country to be weighted and converted into exporter-importer-time level data for regression. The PCT patent data is obtained from the World Intellectual Property Organization (WIPO). The remaining variables were the same as in model (1), and the fixed effects of exporter-year and importer-year were also fixed in models (5) and (6).

The regression results in Table 12 showed that the regression coefficients of the depth of digital intellectual property rules in RTAs were all significantly positive, indicating that it can promote the expansion of two-way FDI in exporting countries. Strengthening the level of intellectual property protection can promote both OFDI [57] and IFDI [58]. It is an important manifestation for improving the level of intellectual property protection in contracting countries to sign RTAs with digital intellectual property protection rules, and the outward direct investment behavior of knowledge-intensive enterprises will be encouraged, which is conducive to enhancing the scale of a two-way FDI in exporting countries. At the same time, according to the theoretical analysis above, the expansion of two-way FDI scale in exporting countries is conducive to promoting the export technology complexity. Therefore, the digital intellectual property rules in RTAs can improve the export technology complexity of digital service enterprises through the two-way FDI scale expansion effect of exporting countries.

**Table 12:** Mechanism test of FDI.

Variable	(1)	(2)	(3)	(4)
	lnFDI	lnFDI	lnFDI	lnFDI
lnDepth	0.2718***			
	(0.0340)			
$ln{Depth}^{\text{1}}$		0.2990***		
		(0.0478)		
$ln{Depth}^{\text{2}}$			0.4366***	
			(0.0484)	
$ln{Depth}^{\text{3}}$				0.3192***
				(0.0382)
Constant	−39.7549***	−37.8089***	−38.8955***	−40.2191***
	(13.1000)	(13.3937)	(13.0474)	(13.0824)
Exporter-year fixed effect	Yes	Yes	Yes	Yes
Importer-year fixed effect	Yes	Yes	Yes	Yes
Observation	3,804	3,804	3,804	3,804
R^2^	0.6144	0.6105	0.6172	0.6152

Note: *, **, and *** indicate significant at the 10%, 5%, and 1%, respectively. Values in parentheses are robust standard errors.

In Table 13, the regression results showed that the regression coefficients of the depth of digital intellectual property rules were all significantly positive at the level of 1%, indicating that it helped to improve the technological innovation level of exporting countries. The intellectual property system was conducive to protecting the achievements of intellectual creation, ensuring the investment return and benefits of innovation subjects, and then encouraging enterprises to carry out innovative activities [59]. The higher the commitment level of digital intellectual property rules in RTAs, the better the institutional environment for export enterprises to research and develop new technologies. Innovators are more willing to implement invention and creation activities and apply for patent protection for new technologies, and the technological innovation level of export enterprises can be improved. At the same time, according to the above theoretical analysis, the improvement of the level of technological innovation in exporting countries is conducive to promoting the export technology complexity. Therefore, the rules of digital intellectual property rules in RTAs can improve the export technology complexity of digital service enterprises through the technological innovation effect of exporting countries.

**Table 13:** Mechanism test of technological innovation.

Variable	(1)	(2)	(3)	(4)
	lnINNO	lnINNO	lnINNO	lnINNO
lnDepth	0.1992***			
	(0.0288)			
$ln{Depth}^{\text{1}}$		0.2160***		
		(0.0409)		
$ln{Depth}^{\text{2}}$			0.3295***	
			(0.0413)	
$ln{Depth}^{\text{3}}$				0.2367***
				(0.0322)
Constant	−32.9507***	−31.5652**	−32.2604***	−33.2786***
	(11.9710)	(12.2900)	(11.8923)	(11.9546)
Exporter-year fixed effect	Yes	Yes	Yes	Yes
Importer-year fixed effect	Yes	Yes	Yes	Yes
Observation	3,804	3,804	3,804	3,804
R^2^	0.7486	0.7465	0.7505	0.7492

Note: *, **, and *** indicate significant at the 10%, 5%, and 1%, respectively. Values in parentheses are robust standard errors.

## Conclusions and recommendations

Based on the export technology complexity of bilateral digital service trade of 64 economies around the world, this paper uses the extended gravity model and two-way fixed effect estimation to attempt to explain the heterogeneous impact of RTAs on trade from the perspective of digital intellectual property rules. The main conclusions are as follows: Firstly, the higher the commitment level of digital intellectual property rules in RTAs, the more conducive it is to improving the export technology complexity of bilateral digital service trade. Through strengthening intellectual property protection, optimizing the trade environment and promoting technological upgrading, the high-quality development of bilateral digital service trade can be effectively promoted. Secondly, the results of heterogeneity analysis show that both digital technology protection rules and digital intellectual property enforcement protection rules significantly promote the export technology complexity of bilateral digital service trade, but the impact of digital copyright protection rules on export technology complexity is not significant. The depth of digital intellectual property rules can significantly increase the export technology complexity of digital service trade in developed countries. The stronger the imitation ability of importing countries, the greater the promotion effect of digital intellectual property rules on the export technology complexity of digital service trade. The greater the gap in law enforcement between import and export countries, the more high-standard digital intellectual property rules can promote the improvement of the export technology complexity of digital service trade. Based on the heterogeneous impact of digital intellectual property rules in RTAs on the technological complexity of digital service export trade, different types of rules can be contracted and improved in a targeted manner to strengthen the protection of digital intellectual property rights on both sides of the trade, thus promoting the technological upgrading of digital service enterprises and enhancing the technological complexity of exports. Thirdly, the results of the mechanism test show that the digital intellectual property rules in RTAs can improve the export technology complexity of digital service enterprises through the two-way FDI scale expansion effect and technological innovation effect of exporting countries. Therefore, utilizing these two effects to improve the policy support and regulatory system can further promote the international competitiveness enhancement of digital service enterprises and the improvement of export technological complexity.

The policy implications of this paper are as follows: First, countries around the world should further promote institutional openness, promote the conclusion of RTAs, and actively promote the further updating and upgrading of existing RTAs. High-standard rules, such as digital intellectual property rules, should be added to RTAs to enhance the depth of RTAs, and then further improve global economic and trade rules and digital intellectual property governance framework. Specifically, policymakers can draw on international high-standard agreements, such as the CPTPP, to build a system of digital intellectual property rules. It can meet their own national conditions and the international situation, and maximize the trade effects of digital intellectual property rules. Second, introduce more relevant rules on digital technology protection and digital intellectual property law enforcement protection in the negotiation of RTAs, such as source code protection and Internet service provider responsibility, to give full play to the trade effect of RTAs and promote the high-quality development of global digital service trade. Third, under the background that RTAs have become the main carrier of global trade rules reconstruction, countries should focus on formulating differentiated negotiation strategies for the digital intellectual property rules of RTAs according to the differences in economic development level, imitation ability and law enforcement of different contracting parties, and then put forward negotiation proposals in line with their own interests. For example, developed countries can consider accepting stricter digital intellectual property protection measures in exchange for more market access and technical cooperation opportunities, whiles developing countries need to balancing the protection of digital intellectual property rights with their own development needs. Fourth, in the process of vigorously developing digital service trade, countries should emphasize the importance of intellectual property protection to improve the quality of service trade, improve relevant laws and regulations on intellectual property protection in the field of domestic rules, thus promoting the integration and docking of domestic and international rules. Due to the limitations of data acquisition, the measurement of this study of digital intellectual property rules relies mainly on the TAPED database. In subsequent studies, other measurement methods can be further explored and the breadth of digital intellectual property rules can be further incorporated into the study.

## Supporting information

S1 Data All variables used in empirical testing. (XLSX)
